# Genomic analysis of antimicrobial resistance and virulence among gram-negative bloodstream isolates from Lebanon

**DOI:** 10.1128/spectrum.00503-26

**Published:** 2026-06-17

**Authors:** Carni Boujanian, Charbel Al Khoury, Mira El Chaar, Rindala Saliba, Lydia-Rossa Hanna, Sima Tokajian

**Affiliations:** 1 Department of Biological Sciences, Lebanese American University, Byblos Campus 114792 , Byblos, Lebanon; 2 Department of Biological Sciences, Lebanese American University, Beirut Campus 37607, Beirut, Lebanon; 3 Faculty of Health Sciences, University of Balamand 66980, Beirut, Lebanon; 4 Faculty of Medicine, Saint Joseph University of Beirut 36925, Beirut, Lebanon; Zhejiang University School of Medicine Sir Run Run Shaw Hospital, Hangzhou, Zhejiang, China

**Keywords:** bloodstream infections, gram-negative bacteria, antimicrobial resistance, carbapenem-resistant* Enterobacterales*, extended-spectrum β-lactamases, mobile genetic elements, virulence factors

## Abstract

The increasing prevalence of multidrug-resistant (MDR) gram-negative bacteria has reduced therapeutic options for bloodstream infections (BSIs) and complicates clinical management. In Lebanon, limited genomic surveillance has restricted insight into the molecular determinants underlying resistance and virulence in invasive pathogens. In this study, whole-genome sequencing was used to characterize antimicrobial resistance, mobilome composition, and virulence gene content among 24 gram-negative isolates recovered from BSIs. Six species were identified, including *Escherichia coli*, *Klebsiella pneumoniae*, *Citrobacter farmeri*, *Citrobacter portucalensis*, *Proteus mirabilis*, and *Morganella morganii*. Phenotypic testing showed widespread resistance to β-lactam and non-β-lactam antimicrobial classes. Extended-spectrum β-lactamase production was detected in 62.5% of isolates, and 37.5% were classified as carbapenem-resistant Enterobacterales. Genomic analyses revealed that resistance in *E. coli* and *K. pneumoniae* was largely driven by plasmid-mediated ESBL and carbapenemase genes associated with IncF replicons and insertion sequences, whereas chromosomal mechanisms predominated in *Citrobacter* species. A chromosomally encoded *bla*_OXA-244_ variant was identified in an *E. coli* ST69 isolate, and a high-risk *K. pneumoniae* ST147 isolate carried multiple β-lactamase genes across distinct plasmids. Colistin resistance in one *K. pneumoniae* isolate was associated with a frameshift mutation in *mgrB* in the absence of plasmid-mediated *mcr* genes. Virulence profiling showed heterogeneous repertoires across species, with expanded adhesion, iron acquisition, and serum resistance determinants in *E. coli*. This study provides the first genome-level characterization of *C. farmeri* and *C. portucalensis* BSIs in Lebanon and supports the integration of genomic surveillance into routine monitoring of invasive infections.

**IMPORTANCE:**

This study presents a pilot whole-genome sequencing analysis of 24 gram-negative bloodstream isolates from a tertiary care hospital in Lebanon. By integrating phenotypic susceptibility testing with genomic, mobilome, and virulence analyses, it reveals marked species- and isolate-level diversity. Resistance and virulence determinants were found within specific genetic contexts rather than being uniformly linked to species or sequence types, underscoring the value of genome-based surveillance to inform antimicrobial stewardship and infection control strategies.

## INTRODUCTION

Bloodstream infections (BSIs) are among the most severe infectious syndromes and are frequently associated with progression to sepsis, resulting in substantial morbidity and mortality. BSIs are defined by the presence of microbial pathogens, most commonly bacteria and less frequently fungi, in the bloodstream and represent a major burden on health care systems worldwide. In 2017, sepsis, often precipitated by BSIs, was estimated to contribute to approximately 11 million deaths, accounting for nearly 20% of all global mortality (1). Although not all BSIs progress to sepsis, the risk of adverse outcomes remains high, particularly among hospitalized, critically ill, and immunocompromised patients. Recent studies indicate that in-hospital mortality associated with sepsis may exceed 30%, depending on the causative pathogen and antimicrobial resistance profile (2).

Bacteremia, which constitutes most BSIs, accounts for more than 90% of reported cases and occurs in both community and health care settings (3). Clinical manifestations range from mild, nonspecific symptoms to severe systemic illness and organ dysfunction. A broad spectrum of pathogens can cause BSIs, with *Escherichia coli* and *Staphylococcus aureus* reported most frequently at the global level. However, the distribution of BSI pathogens varies across regions, influenced by antimicrobial prescribing practices, infection control measures, and local health care infrastructure. Importantly, the rise in antimicrobial resistance (AMR) among BSI pathogens has emerged as a major global health concern, complicating treatment and contributing to poor clinical outcomes (4, 5).

In recent years, the epidemiology of BSIs has shifted toward a predominance of MDR gram-negative organisms in many regions. Particularly in low- and middle-income countries, gram-negative bacteria such as *E. coli*, *Klebsiella pneumoniae*, *Pseudomonas aeruginosa*, and *Acinetobacter baumannii* have become increasingly prominent causes of BSIs. A nationwide study from South Korea identified *E. coli* as the leading BSI pathogen, followed by *S. aureus*, *K. pneumoniae*, and *A. baumannii*, reflecting the growing contribution of gram-negative organisms to invasive infections (6). These pathogens frequently exhibit resistance to multiple antimicrobial classes, including β-lactams and carbapenems, largely driven by the acquisition of resistance genes carried on mobile genetic elements such as plasmids and insertion sequences. The dissemination of these elements has complicated empirical therapy and increased reliance on last-resort agents, including colistin (4).

In Lebanon, genomic data on the molecular characteristics of BSI-causing pathogens remain rare, with limited genomic epidemiology, plasmid tracking, or detailed carbapenemase data. Existing studies have largely focused on phenotypic resistance trends and clinical outcomes. For example, hospital-based surveillance reported a reduction in *A. baumannii* and difficult-to-treat resistant infections during the COVID-19 pandemic (7), while other investigations highlighted high blood culture contamination rates and inappropriate antimicrobial use (8). Among patients with hematological malignancies, *E. coli* was identified as the most common cause of BSIs, with a high proportion of isolates producing extended-spectrum β-lactamases and exhibiting MDR (9). However, these studies relied primarily on routine microbiological methods and did not incorporate whole-genome sequencing (WGS) or detailed analysis of resistance gene contexts.

Although limited in size, this pilot study provides a foundational genomic snapshot of 24 gram-negative bacterial isolates recovered from BSIs at a tertiary care hospital in Lebanon. This study represents one of the first genome-based investigations of BSI pathogens in the country. The objectives were to characterize antimicrobial resistance determinants, with an emphasis on β-lactamases and carbapenemases, and to examine their genetic contexts, including the contribution of insertion sequences and other mobile genetic elements. In parallel, we assessed virulence gene content and integrated these findings with phenotypic susceptibility data. Virulence gene analysis in BSIs is critical for linking genetic determinants of pathogenicity with clinical outcomes, improving as a result our understanding of bacterial invasiveness and disease severity. Through this combined approach, the study aims to provide insight into the genomic diversity of BSI-associated gram-negative pathogens and to support improved antimicrobial stewardship and infection control strategies in the local clinical setting.

## MATERIALS AND METHODS

### Bacterial isolate collection and distribution

Gram-negative bacterial isolates associated with BSIs were obtained from positive blood cultures processed during routine clinical diagnostics at Hôtel-Dieu de France University Hospital, Beirut, Lebanon. Only non-duplicate isolates from individual patients were included to avoid repeated sampling from the same patient. The isolates were consecutively collected as part of routine diagnostic activity during the study period in 2022. From this collection, a subset of 24 MDR bloodstream isolates representing different bacterial species and antimicrobial resistance phenotypes was selected for whole-genome sequencing and subsequent genomic analysis. The isolates were assigned unique identifiers based on the following scheme: *Escherichia coli* (Ec1–Ec3, Ec5, Ec7–Ec12, and Ec14–Ec18), *Klebsiella pneumoniae* (Kp1–Kp5), *Proteus mirabilis* (Pm), *Morganella morganii* (Mm), *Citrobacter portucalensis* (Cp), and *Citrobacter farmeri* (Cf).

### Clinical and demographic data collection

Clinical and demographic data associated with the sequenced isolates were retrospectively retrieved from hospital medical records. The collected metadata included patient sex, age, hospital admission unit, reason for admission, presence of invasive devices (urinary catheter and central venous line), surgical procedures, and documented infections during hospitalization when available. These variables were recorded for each isolate and are summarized in Table 1. Due to the retrospective nature of the data set, complete clinical information was not available for all patients, and classification of infections as community-acquired or hospital-acquired could not be consistently determined.

**TABLE 1:** Demographic and clinical characteristics of patients with carbapenem-resistant Enterobacterales isolates^*a*^

Isolate	Organism	Sex	Age	Admission unit	Reason for admission	Invasive procedures	Reported infection during hospitalization
Ec 1	*E. coli*	F	62	Gynecology	NA	NA	NA
Ec 2	*E. coli*	F	90	Hemato-Oncology	Hemodynamic instability	No	UTI
Ec 3	*E. coli*	F	62	Hemato-Oncology	Gastrointestinal symptoms	Urinary catheter, surgery during hospitalization	No
Ec 5	*E. coli*	M	53	Dialysis/Nephrology	Urinary tract infection	No	No
Ec 7	*E. coli*	F	71	Hemato-Oncology	Respiratory symptoms	Central line, surgery during hospitalization	No
Ec 8	*E. coli*	F	60	Hemato-Oncology	Gastrointestinal symptoms	No	No
Ec 9	*E. coli*	M	53	Hemato-Oncology	Hemodynamic instability	No	UTI
Ec 10	*E. coli*	M	81	Nephrology	Gastrointestinal symptoms	Urinary catheter, surgery during hospitalization	UTI
Ec 11	*E. coli*	F	54	Hemato-Oncology	Hemodynamic instability	No	UTI
Ec 12	*E. coli*	M	72	Hemato-Oncology	Hemodynamic instability	Central line	UTI
Ec 14	*E. coli*	F	93	Nephrology	Hemodynamic instability	No	No
Ec 15	*E. coli*	M	77	Nephrology	Gastrointestinal symptoms	No	UTI
Ec 16	*E. coli*	M	79	Cardiology	Cardiac symptoms	No	UTI
Ec17	*E. coli*	M	69	Hemato-Oncology	Chemotherapy	No	No
Ec18	*E. coli*	M	56	Hemato-Oncology	Gastrointestinal symptoms	No	No
Kp 1	*K. pneumoniae*	F	66	Infectious Diseases	NA	NA	NA
Kp 2	*K. pneumoniae*	M	75	Nephrology	Respiratory symptoms	Urinary catheter, surgery during hospitalization	No
Kp 3	*K. pneumoniae*	F	71	Emergency Department	Respiratory symptoms	No	No
Kp 4	*K. pneumoniae*	M	64	Cardiology	Cardiac symptoms	No	RI
Kp 5	*K. pneumoniae*	M	40	Hemato-Oncology	Chemotherapy	No	No
Cp	*C. portucalensis*	M	32	Infectious Diseases	NA	NA	NA
Cf	*C. farmeri*	M	28	ICU	Urinary tract infection	Central line	No
Mm	*M. morganii*	F	70	Emergency Department	NA	NA	NA
Pm	*P. mirabilis*	M	84	ICU	Hemodynamic instability	No	No

^*a*^ ICU, intensive care unit; NA, missing data; RI, respiratory infection; UTI, urinary tract infection.

### Antimicrobial susceptibility testing

#### Disk diffusion

Antimicrobial susceptibility testing was performed using the Kirby-Bauer disk diffusion method, with each isolate tested once. Fresh bacterial cultures were suspended in sterile 0.9% saline and adjusted to the 0.5 McFarland turbidity standard. The standardized suspensions were inoculated onto cation-adjusted Mueller-Hinton (CAMH) agar plates, and antibiotic discs were placed on the surface. The following antibiotics and disc concentrations (µg) were tested: amikacin (30), gentamicin (10), kanamycin (30), amoxicillin (25), ampicillin (10), amoxicillin-clavulanic acid (20/10), piperacillin-tazobactam (100/10), cefuroxime (30), ceftazidime (30), cefixime (5), cefotaxime (30), cefepime (30), cefoxitin (30), levofloxacin (5), norfloxacin (10), ofloxacin (5), ciprofloxacin (5), tetracycline (30), imipenem (10), meropenem (10), ertapenem (10), and trimethoprim-sulfamethoxazole (1.25/23.75). Plates were incubated at 35 ± 2°C for 16–18 h, and inhibition zone diameters were measured. A quality control strain of *E. coli* ATCC 25922 was included to ensure the reliability of the results. Results were interpreted according to the Clinical and Laboratory Standards Institute (CLSI) guidelines (10).

#### Colistin broth microdilution (BMD)

Minimum inhibitory concentrations (MICs) of colistin (polymyxin E) for three isolates (Kp4, Pm, and Mm) were determined using the reference broth microdilution (BMD) method, performed in triplicate to ensure reproducibility, according to the European Committee on Antimicrobial Susceptibility Testing (EUCAST) guidelines, version 2021 (11). Colistin stock solutions were prepared at 1 mg/mL and diluted in cation-adjusted Mueller-Hinton broth (MH II; Sigma-Aldrich, Merck KGaA, Germany) using twofold serial dilutions to achieve a final concentration range of 0.125 to 64 μg/mL. The assay was conducted in 96-well, non-treated polystyrene microplates. A bacterial suspension was prepared by growing each isolate overnight at 37 °C on Luria-Bertani (LB) agar. A well-isolated colony was transferred into 5 mL MH II broth and incubated at 37 °C until a turbidity equivalent to the 0.5 McFarland standard was reached. A final inoculum of 5 × 10⁵ CFU/mL was added to each well containing colistin. Positive and negative controls were included in each assay: the positive control consisted of MH II broth with bacterial inoculum, while the negative control contained broth without inoculum. *E. coli* ATCC 25922 was used as a quality control strain to validate test performance. Plates were incubated at 37°C for 18–20 h and visually examined under bright, indirect light against a dark background. MICs were defined as the lowest concentration of colistin that completely inhibited visible bacterial growth. According to EUCAST breakpoints, isolates with MICs ≤ 2  µg/mL were considered susceptible, and those with MICs > 2  µg/mL were considered resistant.

#### DNA extraction

Fresh bacterial cultures were grown on tryptic soy agar (TSA) plates (Bio-Rad, USA) at 37°C for 18–24 h. DNA extraction was performed using the Sigma-Aldrich DNA extraction kit, following the manufacturer’s instructions. The extracted DNA was stored at −20°C for short-term use and at −80°C for long-term storage and downstream analyses.

#### Plasmid replicon typing

Plasmid typing was conducted using the DIATHEVA PCR-Based Replicon Typing (PBRT) kit (Diatheva, Fano, Italy), which targets 30 common plasmid replicons found in Enterobacteriales. These include HI1, HI2, I1, I2, X1, X2, X3, X4, L, M, N, FIA, FIB, FIC, FII, FIIS, FIIK, FIB KN, FIB KQ, W, Y, P1, A/C, T, K, U, R, B/O, HIB-M, and FIB-M (12). The kit utilizes eight multiplex PCR assays, each containing a specific positive control. PCR amplification was carried out according to the manufacturer’s instructions. Amplicons were separated by electrophoresis on a 2.5% agarose gel stained with ethidium bromide and visualized after running for 40 min at 80 V.

### Whole-genome sequencing

#### Library preparation

DNA libraries were prepared using the Illumina Nextera XT DNA library preparation kit (Illumina) according to the manufacturer’s protocol. Genomic DNA (gDNA) was subjected to tagmentation, followed by amplification with indexed primers to incorporate sample-specific barcodes and adaptors. Library quantification was performed using a Qubit 2.0 fluorometer (Invitrogen, Carlsbad, CA, USA). The libraries were then normalized, pooled, and loaded onto an Illumina MiSeq system for sequencing using a 2 × 300 bp paired-end protocol, with 30× coverage depth.

#### Quality control, genome assembly, and annotation

The quality of the raw sequencing reads was assessed using FastQC version 0.11.9 (13). *De novo* assembly was performed using SPAdes Genome Assembler (version 3.15.5) with built-in read error correction (14). Quality filtering threshold was Q15, and contig filtering threshold was minimum 200 bp. Genome annotation was conducted using two tools: the Rapid Annotation Subsystem Technology (RAST) server (https://www.bv-brc.org/app/Annotation) and Prokka (Prokaryotic Genome Annotation System), accessed via the Galaxy platform (https://usegalaxy.org).

#### *In silico* genome analysis

Whole-genome analyses were conducted using publicly available tools, primarily from the Center for Genomic Epidemiology (CGE) (https://www.genomicepidemiology.org/). Species identification was confirmed using KmerFinder v3.1, a k-mer-based genome comparison tool (15). Multi-locus sequence typing (MLST) was performed *in silico* using both the CGE MLST tool (https://cge.food.dtu.dk/services/MLST/) with 90% identity threshold and 60% minimum coverage, and the PubMLST database, when applicable. *E. coli* isolates were assigned to phylogroups using the Clermontyping tool (http://clermontyping.iame-research.center/).

Antibiotic resistance genes (ARGs) were identified using ResFinder v4.1 (https://genepi.food.dtu.dk/resfinder), with 90% identity threshold and 60% minimum coverage, and the Comprehensive Antibiotic Resistance Database (CARD) (16). Virulence genes were detected using VirulenceFinder v2.0 (17, 18), with 90% identity threshold and 60% minimum coverage. Insertion sequences (ISs) were identified using ISfinder (19), and other mobile genetic elements (MGEs) were analyzed using CGE tools.

Plasmid replicons were identified using PlasmidFinder v2.1, with 95% identity threshold and 60% minimum coverage. Plasmid contigs were further assembled using plasmidSPAdes v3.14.1, annotated with Prokka, and visualized using SnapGene Viewer (snapgene.com).

#### Biofilm formation

Biofilm formation was assessed using the 96-well microtiter plate method (20), with minor modifications. Fresh bacterial cultures were grown overnight on TSA plates at 37 °C, then inoculated into Luria-Bertani (LB) broth supplemented with 2% sterile-filtered glucose and incubated overnight at 37 °C. Cultures were diluted 1:100 in the same LB-glucose medium, and 100 μL of each dilution was transferred into six wells of a flat-bottomed 96-well microtiter plate, followed by incubation at 37 °C for 24 h. After incubation, the plates were gently inverted, washed thoroughly with distilled water to remove non-adherent cells, and stained with 125 μL of 0.1% crystal violet for 10 min. Excess stain was removed, and the wells were washed multiple times with distilled water before air-drying in an inverted position at room temperature for 24 h. To solubilize the retained dye, 125 μL of 30% acetic acid was added to each well and incubated for 10–15 min at room temperature. Optical density (OD) was measured at 570 nm using a Multiskan FC Microplate Photometer (Thermo Scientific, USA), and results were analyzed using SkanIt software. Isolates were classified into four biofilm formation categories based on the formula (OD: Optical Density, OD_cut_: Optical Density Cut, O_Dav_ Optical Density average of 3 × 2 replicates of the negative control, SD: Standard Deviation):

OD_cut_ = OD_av_+ 3 × SD. ODₐᵥ represents the average optical density of three negative control replicates, and SD is the standard deviation. Classification was as follows: Non-former: OD < OD_av_, Weak former: OD_av_ < OD < 2 × OD_av_, Moderate former: 2 × OD _av_< OD < 4 × OD_av_, Strong former: OD > 4 × OD_av_.

#### Phenotypic detection of hypermucoviscosity in *Klebsiella pneumoniae*

The string test was performed as a phenotypic screening method to assess the hypermucoviscosity phenotype of *K. pneumoniae* isolates. Fresh bacterial colonies were cultured on TSA and incubated at 37 °C for 18–24 h. A single colony was then gently touched with an inoculation loop and lifted vertically. The formation of a viscous, mucous-like string was observed. Isolates were considered positive for hypermucoviscosity if the string extended to a length of 5 mm or more before breaking. This characteristic is often associated with hypervirulent strains of *K. pneumoniae*, which produce an excessive polysaccharide capsule that contributes to increased pathogenicity and resistance to phagocytosis (21).

#### Pan-genome analysis

Pan-genome analysis was conducted exclusively on *E. coli* isolates, as they represented most of our data set (15/24, 62.5%), supporting more reliable genomic comparisons. Genomes of *E. coli* isolates were first annotated using Prokka (22) to generate GFF3 files required for downstream analysis. Pan-genome analysis was then conducted using the Roary pipeline version 3.12.0 with default parameters (23), and core gene alignments were performed using MAFFT. The following flags were used: roary -e -n -v *.gff. A reference *E. coli* strain Ec K-12 substrain MG1655 (accession number U00096.3) was included. The resulting gene presence/absence matrix and core genome alignments were visualized using Phandango, an interactive web-based tool for phylogenomic exploration (24).

## RESULTS

### Patient demographic and clinical characteristics

The bloodstream isolates analyzed in this study were obtained from 24 individual patients, including 14 males and 10 females, with ages ranging from 28 to 93 years. Patients were admitted to several hospital units, most frequently Hemato-Oncology (*n* = 10), followed by Nephrology (*n* = 5), Cardiology (*n* = 2), Infectious Diseases (*n* = 2), Emergency Department (*n* = 2), ICU (*n* = 2), and Gynecology (*n* = 1). The most common reasons for admission included hemodynamic instability (*n* = 6), gastrointestinal symptoms (*n* = 5), respiratory symptoms (*n* = 3), urinary tract infection (*n* = 2), and cardiac symptoms (*n* = 2). Regarding potential clinical risk factors, urinary catheters were present in three patients, central venous lines in three patients, and surgical procedures during hospitalization were reported in four patients. Documented infections during hospitalization included urinary tract infections (*n* = 7) and one respiratory infection, while no infection was reported or information was unavailable for the remaining cases (Table 1). These 24 BSI isolates were sequenced, and the WGS metrics were documented in Table S2.

### Population structure and pan-genome analysis

#### Pan-genome structure and phylogenetic relationships of *Escherichia coli* isolates

**Fig 1:**
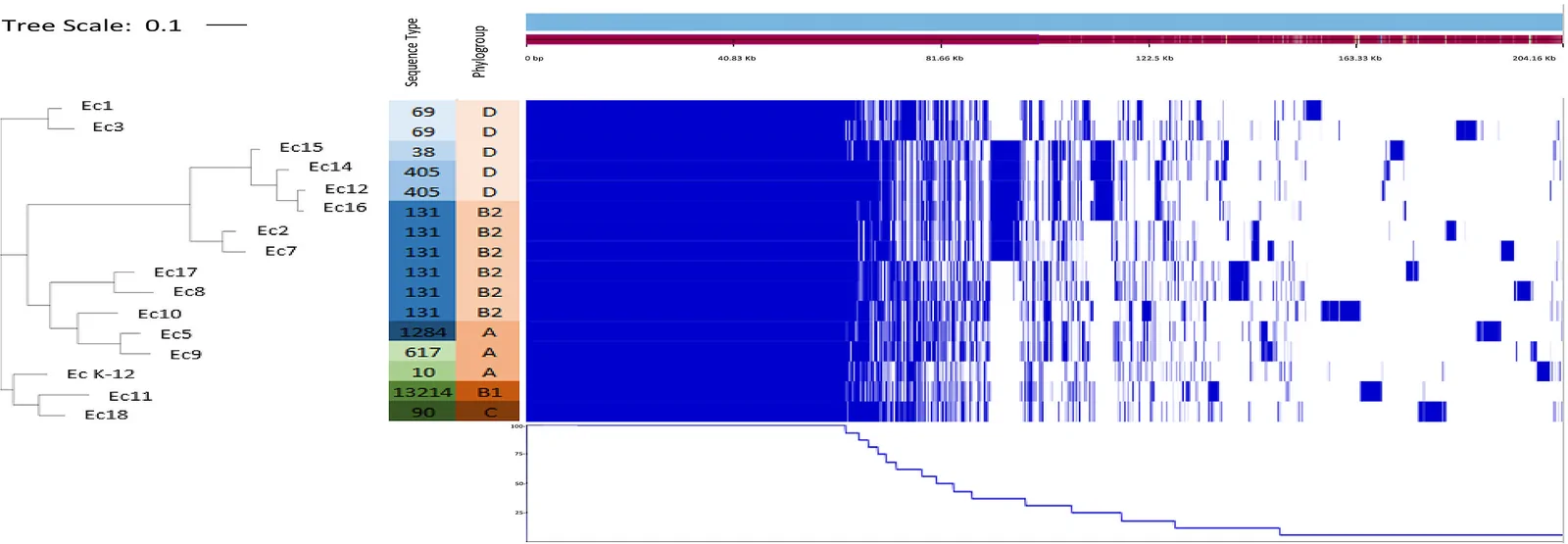
Pan-genome analysis of *Escherichia coli* isolates showing sequence types (STs) and phylogroups. A binary accessory gene tree is shown on the left, and gene presence/absence is displayed on the right. Core genes shared across isolates are represented as continuous blue blocks, while shell and cloud genes appear as dispersed blocks. Colored vertical bars indicate STs and phylogroups, with each color corresponding to a specific classification. The reference strain *E. coli* K-12 MG1655 (accession number U00096.3) is included. The figure was generated using Roary and visualized with Phandango.

Pan-genome analysis (10,206 genes) demonstrated a clear partitioning between a conserved core genome and a highly variable accessory genome among *E. coli* isolates (Fig. 1). A total of 3,142 core genes (30.8%) were consistently present across nearly all isolates, whereas 3,239 shell genes and 3,825 cloud genes exhibited heterogeneous presence–absence distributions, contributing substantially to the observed inter-isolate genomic diversity. Clustering based on accessory gene content indicated that isolates grouped primarily according to sequence type and phylogroup. Isolates belonging to phylogroup B2, dominated by ST131, formed a tight cluster characterized by a shared core genome and overlapping accessory gene profiles, although variability within this group was observed due to differences in accessory gene content. Isolates belonging to phylogroups A, B1, C, and D were more dispersed, reflecting increased genomic diversity.

Isolates assigned to ST131 displayed relatively compact accessory gene patterns compared with non-ST131 lineages. In contrast, isolates from ST69, ST38, ST405, ST617, ST1284, ST13214, and ST90 showed broader distributions of shell and cloud genes and contributed disproportionately to the size of the accessory genome. The accessory gene tree further showed that isolates from different phylogroups often carried distinct gene sets not shared with other lineages, consistent with the contribution of horizontal gene transfer and mobile genetic elements to genomic diversity (Fig. 1).

#### Multi-locus sequence typing (MLST) and phylogrouping

MLST typing of the 15 *E. coli* isolates identified eight distinct sequence types. ST131 was the most prevalent lineage (*n* = 6, 40%), followed by ST69 (*n* = 2, 13.33%) and ST405 (*n* = 2, 13.33%), while ST90, ST13214, ST38, ST617, and ST1284 were each represented by a single isolate (*n* = 1, 6.66%). The five *K. pneumoniae* isolates belonged to distinct sequence types, including ST147, ST1307, ST268, and ST584, with one isolate remaining untypeable. *C. farmeri* was assigned to ST134, while the remaining non-*E*. *coli* isolates could not be assigned to established sequence types.

Phylogrouping of *E. coli* isolates showed a clear association between sequence type and phylogenetic background. All ST131 isolates belonged to phylogroup B2, while ST38, ST69, and ST405 isolates were assigned to phylogroup D. The ST90 isolate (Ec18) belonged to phylogroup C. These assignments were consistent with clustering patterns observed in the pan-genome analysis (Fig. 1), where isolates grouped mainly according to ST and phylogroup.

### Antimicrobial susceptibility and resistance determinants

#### Phenotypic and genomic characterization of non-β-lactam antimicrobial resistance in bloodstream isolates

Phenotypic susceptibility testing showed a high prevalence of resistance to multiple non-β-lactam antimicrobial classes among the bloodstream isolates, including aminoglycosides, fluoroquinolones, tetracycline, and trimethoprim-sulfamethoxazole (Fig. S1). Resistance to kanamycin was observed in 15 out of 24 isolates (62.5%), whereas amikacin retained activity against the majority (*n* = 18, 75%). Fluoroquinolone resistance was widespread, with high rates of resistance to ciprofloxacin (*n* = 19, 79.2%), ofloxacin (*n* = 15, 62.5%), norfloxacin (*n* = 13, 54.2%), and levofloxacin (*n* = 17, 70.83%). Similarly, elevated resistance levels were observed for tetracycline (*n* = 15, 62.5%) and trimethoprim-sulfamethoxazole (*n* = 16, 66.7%) (Fig. S1).

WGS identified resistance determinants that aligned with these phenotypes, including aminoglycoside-modifying enzyme genes (e.g., *aac(3*) and *aad* variants), plasmid-mediated quinolone resistance genes (*qnr* variants), the *oqxAB* efflux system in *K. pneumoniae*, tetracycline efflux genes (*tet*) predominantly in *E. coli*, *Citrobacter*, and *Proteus* spp., and *sul* and *dfrA* genes associated with trimethoprim-sulfamethoxazole resistance (Fig. S2).

Overall, a high level of genotype–phenotype concordance (>95%) was observed across the antimicrobial classes analyzed, indicating strong agreement between genomic predictions and phenotypic resistance profiles. Minor discrepancies were noted, mainly in Kp4, which remained phenotypically susceptible to fluoroquinolones despite harboring resistance-associated genes, potentially reflecting limited gene expression or the presence of non-functional or silent variants.

### Genomic determinants of β-lactam resistance

**Fig 2:**
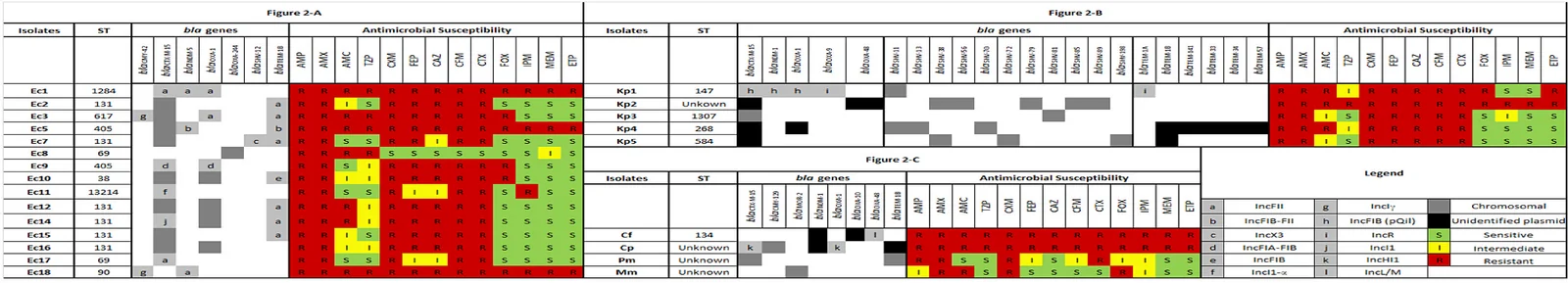
*In silico* detection of β-lactam antibiotic resistance genes and associated plasmid incompatibility groups (identified by PBRT and *in silico* analysis), sequence types (STs), and corresponding Kirby-Bauer disk diffusion susceptibility profiles for the 24 isolates. Antibiotics tested include ampicillin (AMP), amoxicillin (AMX), amoxicillin-clavulanic acid (AMC), piperacillin-tazobactam (TZP), cefuroxime (CXM), cefepime (FEP), ceftazidime (CAZ), cefixime (CFM), cefotaxime (CTX), cefoxitin (FOX), imipenem (IPM), meropenem (MEM), and ertapenem (ETP). Susceptibility categories are defined as S: sensitive, R: resistant, and I: intermediate. bla, β-lactamase gene; Cf, *C. farmeri*; Cp, *C. portucalensis*; Ec, *E. coli*; Kp, *K. pneumoniae*; Mm, *M. morganii*; Pm, *P. mirabilis*.

Distinct β-lactam resistance patterns were observed across the 24 bloodstream isolates (Fig. 2), with overall high genotype–phenotype concordance between the presence of β-lactamase genes and phenotypic resistance profiles. *E. coli* ST131 isolates (Ec2, Ec7, and Ec11–Ec17) consistently carried *bla*_CTX-M-15_, frequently in combination with *bla*_OXA-1_ and *bla*_TEM-1B_, and showed near-complete concordance (>95%) with resistance to penicillins and third-generation cephalosporins, revealing the high sensitivity of these genes for ESBL phenotypes.

The variability in cefepime susceptibility, however, suggests partial discordance, potentially attributable to differential gene expression levels, outer membrane permeability, or other host-related factors. In contrast, non-ST131 *E. coli* isolates, such as Ec8 (ST69), exhibited lower genotype-phenotype concordance. The detection of *bla*_OXA-244_ in this isolate, despite retained susceptibility to advanced cephalosporins and carbapenems, suggests limited functional expression or reduced enzymatic activity associated with this variant.

Sequence type-specific analysis revealed distinct resistance patterns. ST131 (*n* = 6, 40%) consistently showed ESBL-like phenotype with preserved carbapenem susceptibility, whereas carbapenem resistance was confined to non-ST131 isolates (*n* = 3, 33%). Higher resistance burden, including pan-resistance, was observed in ST405, ST90, ST1284, while ST69 retained partial susceptibility. *K. pneumoniae* isolates showed broader β-lactamase repertoires, particularly Kp1 (ST147) and Kp4 (ST268), which carried multiple *bla*_SHV_ variants in addition to *bla*_CTX-M-15_ and were resistant to penicillins and cephalosporins. However, Kp1, despite harboring carbapenem resistance genes, such as *bla*_NDM-1_, was resistant only to ertapenem while remaining susceptible to imipenem and meropenem (Fig. 2), likely due to low-level NDM-1 activity.

*Citrobacter* spp. demonstrated complete genotype–phenotype concordance (100%), as the phenotypic resistance profiles were fully explained by the presence of corresponding resistance genes detected at the genomic level across all tested antimicrobial classes (Fig. 2). In *C. farmeri* the *bla*_OXA-10_ gene was associated with a class 1 integron, while both isolates (Cp and Cf) exhibited resistance to all tested carbapenems, consistent with the presence of *bla*_OXA-48_ and *bla*_NDM-1_.

*P. mirabilis* carrying *bla*_CTX-M-15_ and *bla*_TEM-1B_ was resistant to ampicillin, amoxicillin, cefuroxime, and cefotaxime, intermediate to cefemine, cefexime, cefoxitin, and imipenem, and susceptible to ertapenem, meropenem, ceftazidime, and piperacillin-tazobactam, and amoxicillin-clavulanic acid, yielding a genotype-phenotype concordance of around 62% across tested β-lactams (Fig. 2). This partial concordance suggests that additional factors, such as gene expression levels or regulatory mechanisms, may influence the observed phenotypic susceptibility profile.

Finally, *M. morganii* carrying *bla*_MOR-2_ exhibited resistance to amoxicillin, amoxicillin-clavulanic acid, cefuroxime, and cefoxitin, intermediate resistance to ampicillin and imipenem, and susceptibility to ertapenem, meropenem, cefotaxime, cefixime, ceftazidime, cefepime, and piperacillin-tazobactam. Accordingly, the genotype-phenotype concordance was around 77% across the tested β-lactams. This extended resistance spectrum is likely due to other mechanisms that enhance resistance beyond the narrow-spectrum β-lactamase encoded by *bla*_MOR-2_ alone (Fig. 2).

### Colistin susceptibility and resistance mechanisms

BMD testing identified colistin resistance in *K. pneumoniae* Kp4, *P. mirabilis*, and *M. morganii*, with MICs > 2 µg/mL according to EUCAST breakpoints. Resistance in *P. mirabilis* and *M. morganii* was consistent with intrinsic colistin resistance. In contrast, resistance in *K. pneumoniae* Kp4 was associated with an acquired mechanism. Comparative genomic analysis against the reference strain *K. pneumoniae* MGH78578 (NC_009648.1) identified a thymine deletion at position 20 in the *mgrB* gene, resulting in a frameshift and premature stop codon that inactivated the gene. The observed *mgrB* frameshift is consistent with previously described mechanisms associated with the PhoPQ two-component system and lipid A modification, reducing colistin binding and explaining the observed resistance.

### Mobilome associations with β-lactam resistance

**Fig 3:**
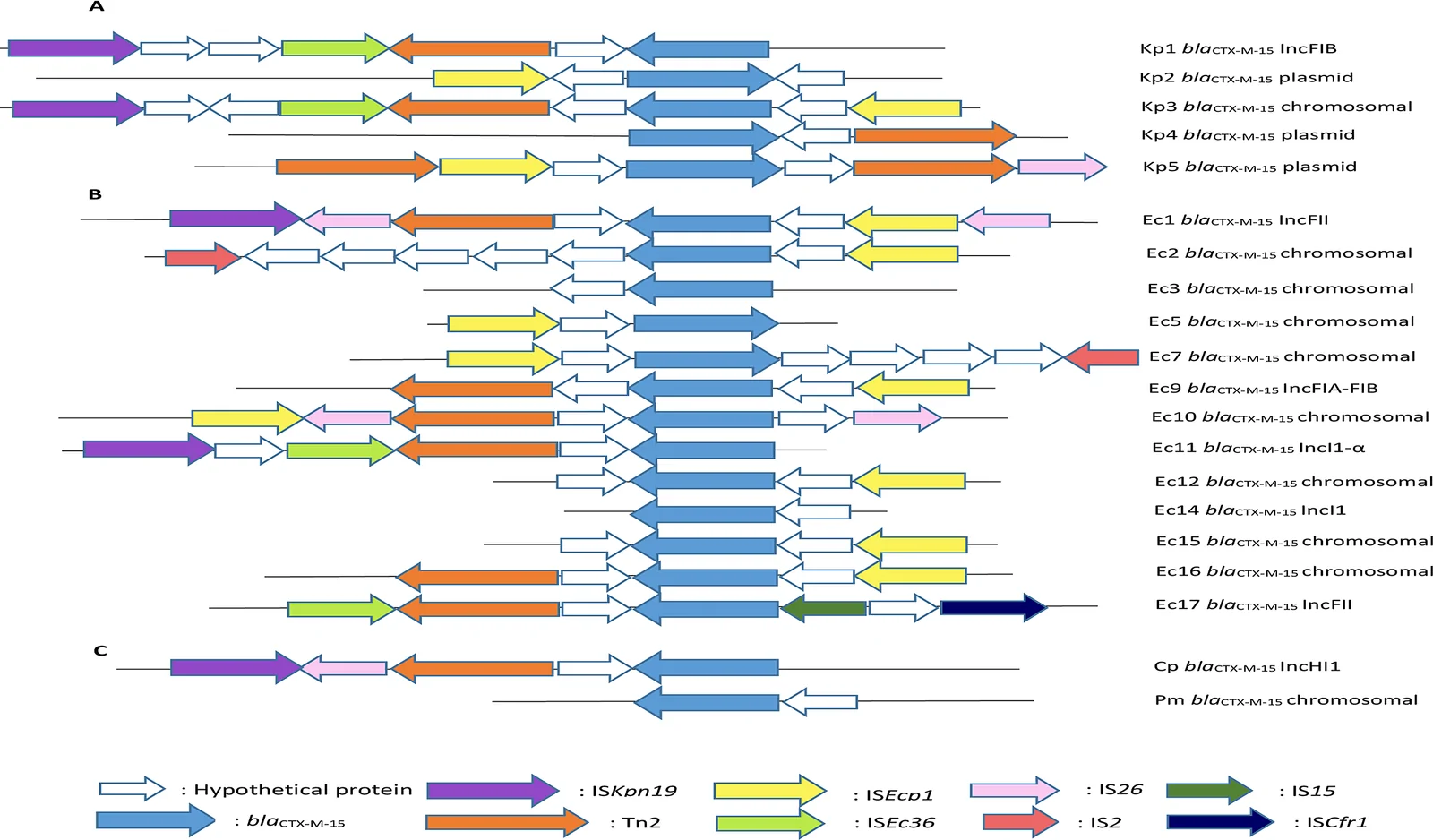
Genetic environments of chromosomal and plasmid-borne *bla*_CTX-M-15_ genes in (**A**) *E. coli*, (**B**) *K. pneumoniae*, and (**C**) *C. farmeri* and *P. mirabilis* isolates. Sequences were annotated using Prokka and visualized through SnapGene Viewer. *bla*_CTX-M-15_, beta-lactamase CTX-M-15-producing gene; IS*2*, IS*3* family transposase IS*2*; IS*26*, IS*6* family transposase IS*26*; IS*Ec36*, IS*3* family transposase IS*Ec36*; IS*Ecp1*, IS*1380* family transposase IS*Ecp1*; IS*Kpn19*, IS*Kra4* family transposase IS*Kpn19*; Tn2, Tn3 family transposase Tn2.

In *E. coli*, β-lactam resistance was frequently associated with plasmid-borne gene clusters, most commonly *bla*_CTX-M-15_ together with *bla*_OXA-1_ and *bla*_TEM-1B_, detected in isolates Ec2, Ec7, and Ec11-Ec17 and mainly located on IncF plasmids, including IncFIB, IncFIA, and IncFII replicons. These clusters were consistently linked to insertion sequences (IS), with IS*Ecp1* identified upstream of *bla*_CTX-M-15_ and IS*26* flanking *bla*_OXA-1_ and *bla*_TEM-1B_. Isolates with higher plasmid burden, such as Ec14, showed increased IS diversity, while *E. coli* isolates with narrower β-lactam resistance profiles, including Ec8 (ST69) and Ec10 (ST38), carried ISs and lacked clustered β-lactamase arrangements (Fig. 3 and 6).

**Fig 4:**
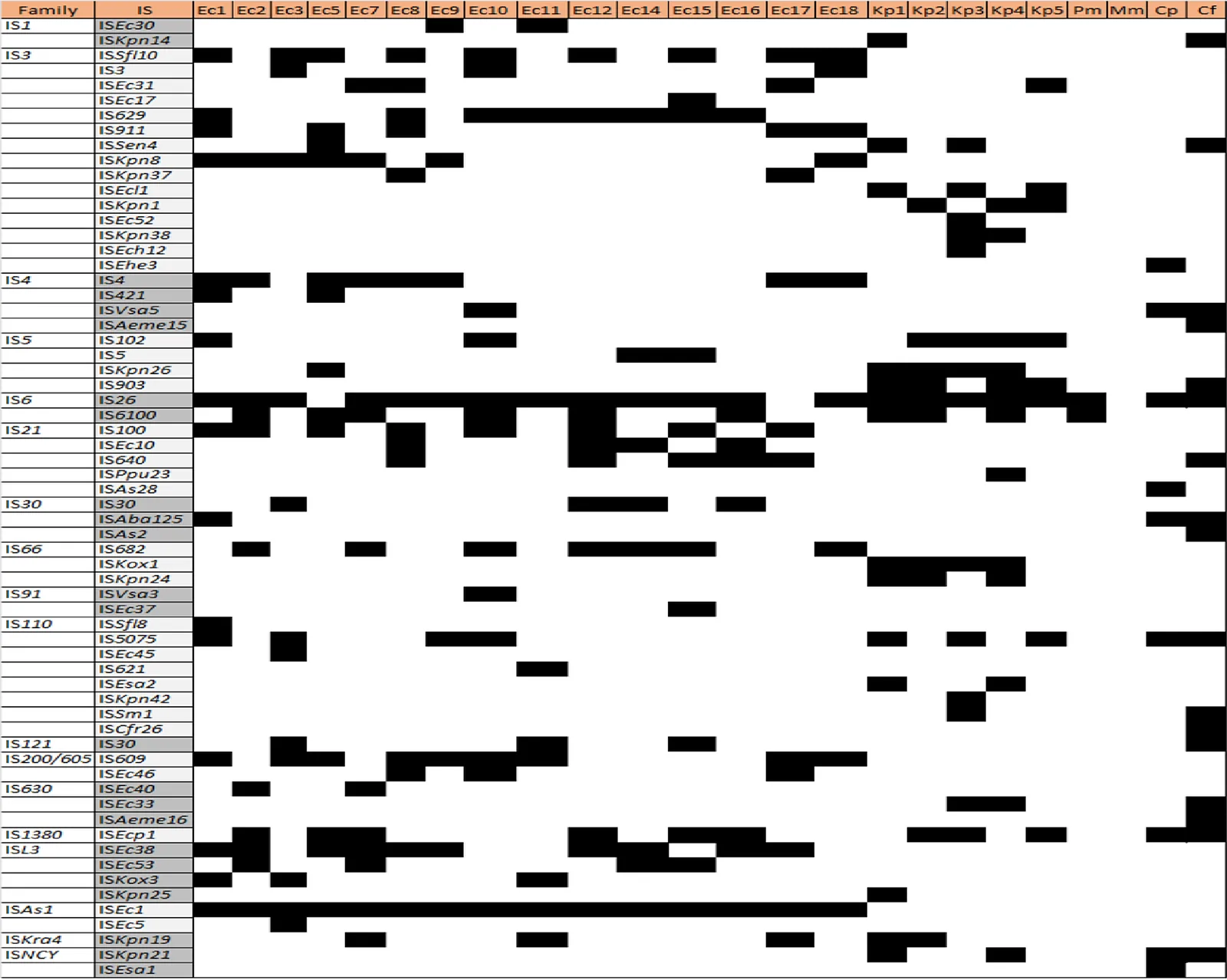
Insertion sequences detected in *E. coli*, *K. pneumoniae*, *P. mirabilis*, *M. morganii*, *C. portucalensis*, and *C. farmeri*, identified using MGE and IS-Finder.

In *K. pneumoniae*, isolates Kp1 (ST147) and Kp4 (ST268) carried a wide range of β-lactamases, including *bla*_CTX-M-15_ in combination with *bla*_SHV_ and *bla*_TEM_ variants, located within IS-rich regions characterized by IS*Kpn* elements, IS*26*, and IS*Ecp1* (Fig. 3). All *K. pneumoniae* isolates carried IS*26*, while additional insertion sequences varied between isolates and corresponded to differences in β-lactamase gene content (Fig. 4). *C. portucalensis* and *C. farmeri* lacked plasmid-associated β-lactamase clusters and IS-flanked ESBL genes, with resistance patterns consistent with chromosomal mechanisms. *P. mirabilis* carried a limited mobilome, including a single IncQ1 plasmid and few insertion sequences, while no insertion sequences were detected in *M. morganii*.

### Virulence determinants

Virulence gene profiling showed marked heterogeneity among the bloodstream isolates, with species- and isolate-level differences (Fig. S3). The mean virulence gene count per species decreased from *E. coli* (19.3) to *K. pneumoniae* (5), *Citrobacter* spp. (2.5), *P. mirabilis* (1), to *M. morganii* (0). In *E. coli*, virulence gene content varied by phylogroup and ST. Phylogroup B2 (ST131; *n* = 6) had a mean of 19.8 genes. Phylogroup D (*n* = 5) showed a lower overall mean (15.6), with variation across STs: ST69 (21), ST38 (13), and ST405 (10). Phylogroup A (*n* = 2) exhibited the highest mean (28.5; ST617: 30, ST1284: 27), while phylogroups B1 (ST13214; 20) and C (ST90; 15) showed intermediate to lower values. These results indicate substantial lineage-associated variation in virulence gene burden, particularly within *E. coli*, whereas non-*E*. *coli* species, as indicated, displayed consistently lower virulence gene counts.

Among *E. coli*, several isolates carried many virulence determinants involving adhesion, iron acquisition, serum resistance, and toxin-associated functions, reflecting strong enrichment across multiple virulence categories. Isolates Ec2, Ec3, Ec7, Ec9, and Ec11-Ec17 had multiple adhesion-related genes, including *fimH, csgA, fdeC*, and *yeh* loci, along with serum resistance-associated genes such as *iss* and *traT*. Iron acquisition systems, including *chuA, iutA, sitA, and fyuA*, were frequently detected. Toxin-associated genes, *hlyE, sat, astA, and senB*, were also present in some of the isolates. In contrast, *E. coli* isolates with narrower resistance and mobilome content, such as Ec8 (ST69) and Ec10 (ST38), carried fewer virulence determinants (Fig. S3).

*K. pneumoniae* isolates showed a moderate mean virulence gene count and a more restricted enrichment profile. Isolates Kp1 (ST147) and Kp4 (ST268) carried iron acquisition genes, including *iutA* and yersiniabactin-related loci, together with capsule-associated determinants such as *cps*- and *kps*-related genes. Fimbrial genes, such as *mrkA*, were detected, while toxin-associated genes were largely absent (Fig. S3).

*C. portucalensis* and *C. farmeri* exhibited a limited virulence gene profile, largely confined to genes associated with stress adaptation, cellular maintenance, and host-associated survival (e.g., *nlpl*, *ter*, and *clpK2*, *shiB*) (Fig. S3).

*P. mirabilis* and *M. morganii* exhibited the lowest mean virulence gene counts, lacking the broad adhesion- and toxin-associated repertoires observed in the other species.

### Hypermucoviscosity (string test)

Phenotypic assessment of hypermucoviscosity among *K. pneumoniae* isolates (Kp1–Kp5) showed that none exhibited a hypermucoviscous phenotype. All isolates produced viscous strings shorter than 5 mm when tested and were classified as negative by the string test.

### Biofilm formation

Biofilm formation assays showed variability among the isolates. *K. pneumoniae* isolates Kp1, Kp2, Kp3, and Kp5 were classified as moderate biofilm formers (4/24, 16.66%), while Kp4 and *P. mirabilis* were weak biofilm formers (2/24, 8.33%). The remaining 18 isolates (75%) did not show detectable biofilm formation under the conditions tested. Genomic analysis showed that all *K. pneumoniae* isolates carried the *mrkA* gene encoding type III fimbriae and the *fimH* gene encoding type I fimbriae, consistent with their observed biofilm-forming capacity.

## DISCUSSION

The global rise of MDR gram-negative bacteria represents a major public health concern, particularly in the context of BSIs, which are associated with substantial morbidity and mortality. In Lebanon, where antimicrobial resistance surveillance remains fragmented, genomic data are essential for understanding the molecular features of invasive pathogens. This study provides an integrated analysis of antimicrobial resistance, mobilome composition, and virulence gene content among bloodstream isolates, revealing clear species- and isolate-level variation. By combining phenotypic susceptibility testing with WGS and mobile genetic element analysis, the data show that resistance and virulence determinants are distributed within specific genetic contexts rather than being uniformly associated with species or STs. Mobile genetic elements play a major role in shaping resistance and virulence profiles in dominant bloodstream pathogens, while chromosomal mechanisms contribute more prominently to species with limited mobilome content.

**Fig 5:**
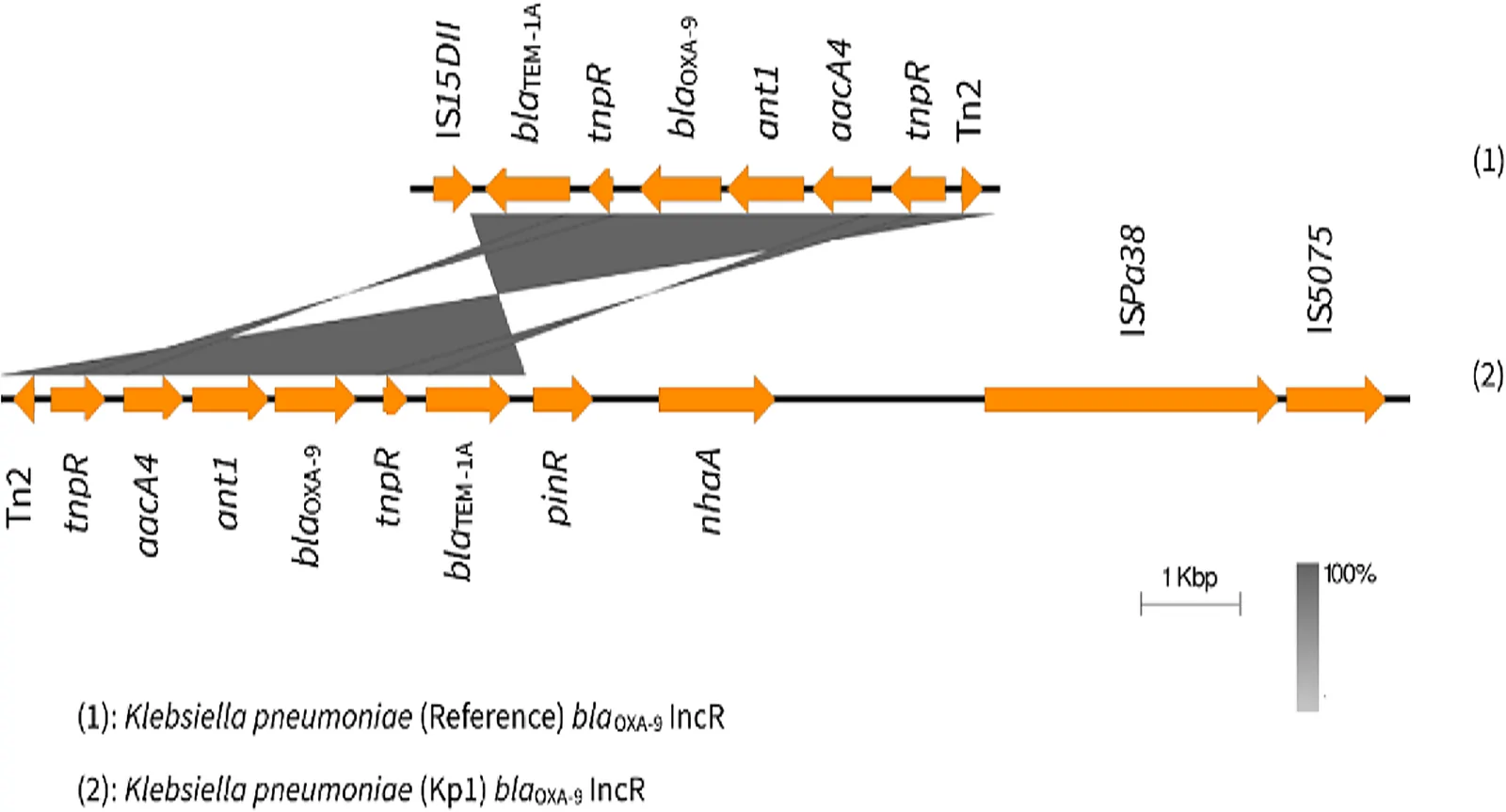
Comparative analysis of the genetic environment of *bla*_OXA-9_, carried by an IncR plasmid in *Klebsiella pneumoniae* (Kp1) and a reference *K. pneumoniae* strain (accession number CP025517.1). this figure was generated using Easyfig. *aacA4*, aminoglycoside N(6′)-acetyltransferase type 1; *ant1*, streptomycin 3′′-adenylyltransferase; *bla*_OXA-9_, beta-lactamase OXA-9-producing gene; *bla*_TEM-1A_, beta-lactamase TEM type 1A; IS*5075*: IS*110* family transposase IS*5075*; IS*Pa38*: Tn3 family transposase IS*Pa38*; nhaA, Na(+)/H(+) antiporter NhaA; pinR, serine recombinase PinR; Tn2: Tn3 family transposase; *tnpR*: Transposon Tn3 resolvase.

A carbapenem-resistant *K. pneumoniae* isolate (Kp1, ST147), as shown in Fig. 2, carrying six β-lactamase genes was identified. *bla*_NDM-1_ and *bla*_CTX-M-15_ were detected on contigs carrying IncFIB replicon sequences, while *bla*_OXA-1_ was located on an IncFIB(pQil) plasmid. *bla*_OXA-9_ and *bla*_TEM-1A_ were carried on an IncR plasmid (Fig. 5), whereas *bla*_SHV-11_ was chromosomally encoded. A related *K. pneumoniae* ST14 strain harboring *bla*_CTX-M-15_, *bla*_DHA-1_, *bla*_TEM-1B_, *bla*_NDM-1_, *bla*_SHV-28_, and *bla*_OXA-1_ was previously reported in Lebanon from a urine sample (25), indicating persistence of similar resistance patterns in the country. IncR plasmids carrying *bla*_OXA-9_ are infrequently described. Comparison of the IncR plasmid identified here with a reference plasmid (CP025517.1) showed a fully conserved MDR region containing *bla*_OXA-9_, flanked by *bla*_TEM-1A_, *tnpR*, *ant1*, *aacA4*, and Tn2-associated elements. While the resistance core was conserved, differences in IS composition, including IS*Pa38* and IS*5075* in our isolate compared with IS*15SII* in the reference plasmid, suggest ongoing structural variation with potential for further rearrangements in clinical settings (Fig. 5).

Another important finding was the detection of chromosomally encoded *bla*_OXA-244_ in an *E. coli* ST69 isolate (Ec8). This OXA-48-like variant shows limited activity against carbapenems and temocillin and remains infrequently reported (26). While *bla*_OXA-244_ has previously been identified in Lebanon, reports involving *E. coli* ST69 remain rare. The gene has been more commonly associated with *E. coli* ST38 in Europe. To date, only a small number of MDR *E. coli* ST69 isolates carrying chromosomal *bla*_OXA-244_ have been described globally, including one reported from Egypt in 2020 in which the gene was embedded within the IS*1R* composite transposon Tn*6237* (27). The present finding expands the limited geographic and clonal documentation of this resistance determinant.

*bla*_CTX-M-15_ was detected in 20 out of 24 isolates (83.33%) and was associated with a diverse set of mobile genetic elements, including IS*Ecp1*, Tn*2*, IS*Kpn11/19*, IS*26*, IS*Ec36*, IS*2*, IS*15*, and IS*Cfr1* (Fig. 3 and 4). Chromosomal integration of *bla*_CTX-M-15_ mediated by IS*Ecp1* has been reported previously, supporting its role in mobilizing resistance determinants between plasmid and chromosomal locations (28). Likewise, studies from Africa reported associations between *bla*_CTX-M-15_ and insertion sequences such as IS*Ecp1* and IS*26* (29). In the present study, IS*Ecp1* and IS*26* were the most frequently detected insertion sequences associated with *bla*_CTX-M-15_, indicating partial overlap with previously described mobilization pathways (Fig. 3 and 4).

**Fig 6:**
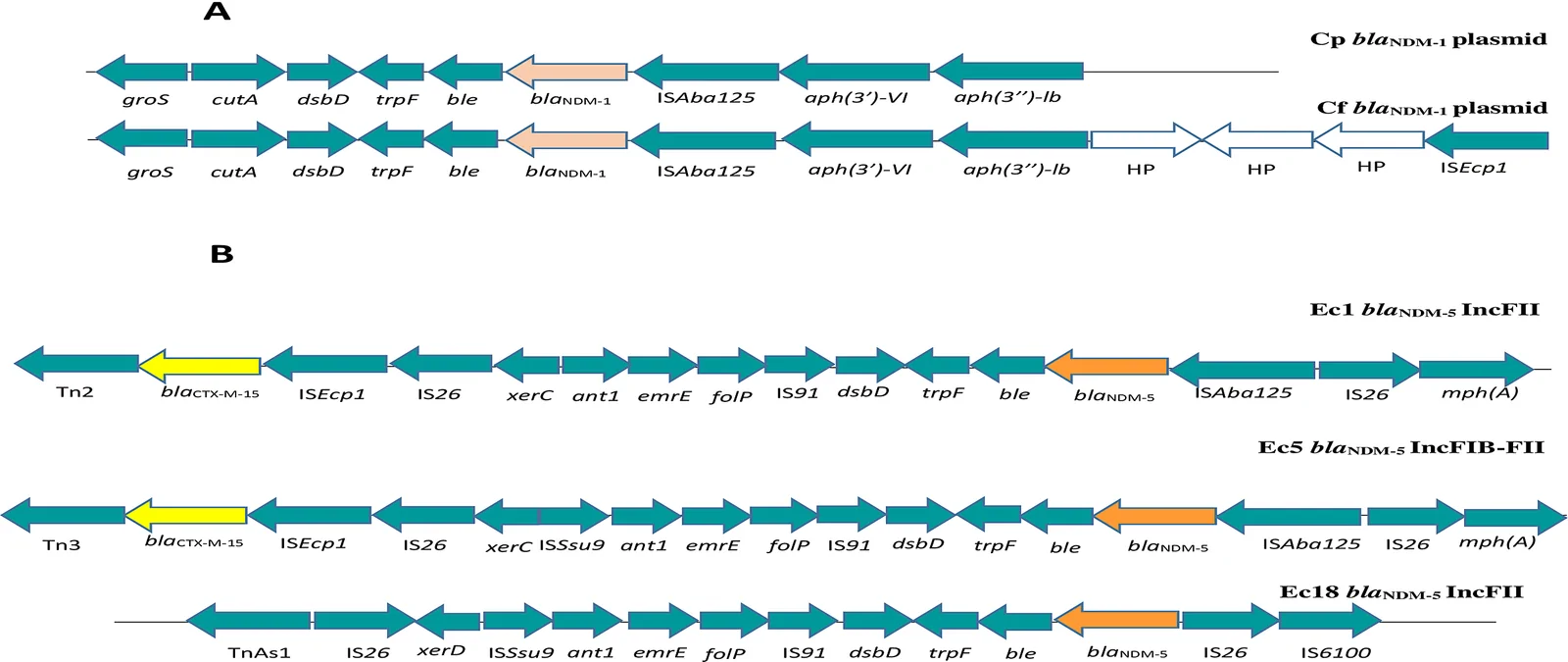
Genetic contexts of plasmid-borne carbapenemase genes. (**A**) *bla*_NDM-1_ in *C. portucalensis* and *C. farmeri*. (**B**) *bla*_NDM-5_ in *E. coli* isolates Ec1, Ec5, and Ec18 isolates. Sequences were annotated using Prokka and visualized with SnapGene Viewer. *ant1*, streptomycin 3*′′*-adenylyltransferase; *aph(3′)-VI*, aminoglycoside phosphotransferase (3′)-VI; *aph(3′′)-Ib*, aminoglycoside phosphotransferase (3″)-Ib; *bla*_CTX-M-15_, beta-lactamase CTX-M-15-producing gene; *bla*_NDM-1_, New Delhi metallo-beta-lactamase-1-producing gene; *ble*, bleomycin resistance protein; *cutA*, divalent-cation tolerance protein CutA; *dsbD*, thiol:disulfide interchange protein DsbD; *emrE*, multidrug transporter EmrE; *folP*, dihydropteroate synthase; *groS*, 10 kDa chaperonin; IS*26*, IS*6* family transposase IS*26*; IS*Aba125*, IS*30* family transposase IS*Aba125*; IS*Ecp1*, IS*1380* family transposase IS*Ecp1*; *mph(A)*, macrolide 2′-phosphotransferase A; Tn2, Tn3 family transposase Tn2; Tn3, Tn3 family transposase; TnAs1, Tn3 family transposase; *trpF*, N-(5′-phosphoribosyl)anthranilate isomerase; *xerC*, tyrosine recombinase XerC..

Carbapenemase gene *bla*_NDM_ was identified in multiple species with distinct genetic contexts. In both *Citrobacter* species, a conserved *groS-cutA-dsbD-trpF-ble-bla*_NDM-1_-IS*Aba125* cassette was identified (Fig. 6), resembling previously reported arrangements in *C. freundii* (30). The absence of IS*CR27* and IS*3000*, together with the presence of additional aminoglycoside resistance genes, suggests lineage-specific remodeling of this resistance cassette. In *E. coli* isolates Ec1, Ec5, and Ec18, *bla*_NDM-5_ was plasmid-borne on IncF replicons and associated with different transposons and ISs, indicating multiple mobilization routes (Fig. 6). Comparable diversity in *bla*_NDM-5_ genetic environments has been reported previously, including localization within novel transposons on IncHI2 plasmids (31).

The *bla*_OXA-10_ gene detected in *C. farmeri* was associated with a class 1 integron, consistent with reports describing integron-linked *bla*_OXA-10_ in *Pseudomonas aeruginosa* and other gram-negative bacteria (32). This finding supports the role of integrons in maintaining and disseminating β-lactam resistance determinants across diverse hosts.

Recent large-scale genomic analyses of *M. morganii* have described extensive resistomes and virulence determinants (33). In contrast, the *M. morganii* isolate recovered in this study lacked acquired carbapenemase or colistin resistance genes and carried only the intrinsic *bla*_MOR-2_ gene (Fig. 2). Its resistance profile was limited to selected β-lactams and colistin, consistent with chromosomal and intrinsic mechanisms rather than acquired resistance.

*P. mirabilis* is recognized for its ability to adhere to surfaces and form biofilms on medical devices (34, 35). Genomic analysis of the isolate recovered here identified genes involved in motility and biofilm formation, including flagellar and fimbrial components, consistent with its observed biofilm-forming phenotype. Prior studies have shown that fimbriae play a key role in adherence to epithelial surfaces (36), while flagella contribute to biofilm development (37).

Colistin resistance remains a growing concern as reliance on last-resort antimicrobials increases. In *K. pneumoniae*, resistance is frequently driven by alterations in two-component regulatory systems or inactivation of *mgrB* (38). Consistent with previously described mechanisms, colistin resistance in Kp4 was associated with *mgrB* inactivation. No plasmid-mediated *mcr* genes were detected, indicating chromosomal adaptation as the primary resistance mechanism, consistent with previous reports (39, 40).

Phylogenetic analysis showed that a substantial proportion of *E. coli* isolates belonged to phylogroup B2 (Fig. 1), which is commonly associated with extraintestinal pathogenic *E. coli* (41). ST131 isolates exhibited high levels of MDR (42). WGS also revealed diverse virulence-linked genes, including bacteriocins, adhesins, and toxins, particularly among ST131 and ST38 isolates, consistent with their association with extraintestinal infections (Fig. S3). The *shiB* gene, a virulence determinant homologous to *Shigella flexneri shiA*, was detected in several *E. coli* isolates and in *C. farmeri* and was associated with the IS*629* mobile element, in agreement with earlier observations in *S. flexneri* (43).

This study also presents the first genome-level characterization of *C. portucalensis* and *C. farmeri* bloodstream isolates in Lebanon. The presence of *bla*_NDM-1_, *bla*_CMY_ variants, and multiple ISs in these rarely reported species highlight their potential to contribute to resistance gene circulation in the clinical setting (Fig. 6).

This study has a small sample size (*n* = 24), reflecting the difficulty of collecting such isolates in Lebanon. Additionally, only short-read WGS was performed. Despite these limitations, this pilot WGS study provides the first comprehensive genomic overview of Gram-negative bacteria causing BSIs in Lebanon. The findings reveal diverse resistance mechanisms, high-risk clonal lineages, and uncommon resistance gene contexts, emphasizing the value of genomic surveillance in this setting. Integration of WGS into routine antimicrobial resistance monitoring would improve detection of emerging resistance, support outbreak investigation, and inform infection control strategies. Future work should incorporate long-read sequencing, functional validation, and expanded sampling to better define resistance dynamics in invasive infections.

## Data Availability

All genomes generated and analyzed in this study have been deposited in the NCBI repository under BioProject accession numbers PRJNA1018810 and PRJNA1018814 (Table S1). The accession numbers of additional reference genomes used are available within the Materials and Methods.

## SUPPLEMENTAL MATERIAL

Fig. S1 spectrum.00503-26-s0001.pdf Kirby-Bauer disk diffusion susceptibility profiles of all the isolates against aminoglycosides (kanamycin, gentamicin, amikacin), fluoroquinolones and related antibiotics (ciprofloxacin, ofloxacin, norfloxacin, tetracycline, levofloxacin), and the sulfonamide trimethoprim-sulfamethoxazole; S: sensitive, R: resistant, I: intermediate. Ec (*E. coli*), Kp (*K. pneumoniae*), Cp (*C. portucalensis*), Cf (*C. farmeri*), Pm (*P. mirabilis*), Mm (*M. morganii*).

Fig. S2 spectrum.00503-26-s0002.pdf In silico detection of antibiotic resistance genes in E. coli (Ec1-Ec3, Ec5, Ec7-Ec12, Ec14-Ec18), K. pneumoniae (Kp1-Kp5), Citrobacter spp. (Cp, Cf), and P. mirabilis (Pm). Resistance gene classes are labeled as follows: A, aminoglycosides; An, antiseptics; C, chloramphenicol; F, fosfomycin; M, macrolides; Q, quinolones; R, trimethoprim; S, sulfonamides; T, tetracyclines.

Fig. S3 spectrum.00503-26-s0003.pdf Virulence determinants detected in *E. coli*, *K. pneumoniae*, *C. portucalensis*, *C. farmeri*, *P. mirabilis*, and *M. morganii* isolates. (Unk: unknown)

Table S1 spectrum.00503-26-s0004.pdf NCBI accession numbers corresponding to the whole-genome sequences of each isolate.

Table S2 spectrum.00503-26-s0005.pdf Genome size (bp), number of contigs ({greater than or equal to}1000 bp), read count, N50 (bp), and GC content (%) are shown for each of the 24 BSI isolates.

ASM does not own the copyrights to Supplemental Material that may be linked to, or accessed through, an article. The authors have granted ASM a non-exclusive, world-wide license to publish the Supplemental Material files. Please contact the corresponding author directly for reuse.
