# Supplementary material for: Genomic analysis of antimicrobial resistance and virulence among gram-negative bloodstream isolates from Lebanon
Source: Microbiol Spectr. 2026 Jun 17;14(7):e00503-26. doi: 10.1128/spectrum.00503-26 (PMC13340248; doi:10.1128/spectrum.00503-26)
Supplement: Fig. S1 — Kirby-Bauer disk diffusion susceptibility profiles of all the isolates against aminoglycosides (kanamycin, gentamicin, amikacin), fluoroquinolones and related antibiotics (ciprofloxacin, ofloxacin, norfloxacin, tetracycline, levofloxacin), and the sulfonamide trimethoprim-sulfamethoxazole; S: sensitive, R: resistant, I: intermediate. Ec (E. coli), Kp (K. pneumoniae), Cp (C. portucalensis), Cf (C. farmeri), Pm (P. mirabilis), Mm (M. morganii). [file spectrum.00503-26-s0001.pdf]

		Isolates																							
		Ec1	Ec2	Ec3	Ec5	Ec7	Ec8	Ec9	Ec10	Ec11	Ec12	Ec14	Ec15	Ec16	Ec17	Ec18	Kp1	Kp2	Kp3	Kp4	Kp5	Pm	Mm	Cp	Cf
Aminoglycoside	Kanamycin	R	S	R	S	S	S	R	R	S	R	R	S	R	I	S	R	R	I	R	S	R	S	R	R
	Gentamicin	R	S	S	S	S	S	S	S	S	R	R	S	S	S	S	S	S	S	R	S	R	S	R	S
	Amikacin	S	S	S	S	S	S	S	S	S	I	I	S	I	S	S	R	R	S	S	S	S	S	R	S
Fluoroquinolones	Ciprofloxacin	R	I	R	R	R	S	R	R	S	R	R	R	R	R	R	R	I	R	S	R	S	S	R	R
	Ofloxacin	R	S	R	R	R	S	R	R	S	R	R	R	R	R	R	R	S	S	S	S	S	S	R	R
	Norfloxacin	R	S	R	R	S	S	R	R	S	R	R	R	R	S	R	R	S	S	S	S	S	S	R	R
	Levofloxacin	R	S	R	R	R	S	R	R	S	R	R	R	R	R	R	R	I	I	S	S	S	S	R	R
Tetracyclin	Tetracyclin	I	R	R	R	R	R	R	R	S	R	S	S	S	R	R	S	S	S	R	R	R	S	R	S
Sulfonamide	Trimethoprim-sulfamethoxazole	S	R	S	R	R	S	R	R	S	R	S	S	R	R	R	R	R	S	R	R	R	S	R	R

Fig. S1 Kirby-Bauer disk diffusion susceptibility profiles of all the isolates against aminoglycosides (kanamycin, gentamicin, amikacin), fluoroquinolones and related antibiotics (ciprofloxacin, ofloxacin, norfloxacin, tetracycline, levofloxacin), and the sulfonamide trimethoprim-sulfamethoxazole; S: sensitive, R: resistant, I: intermediate. Ec (*E. coli*), Kp (*K. pneumoniae*), Cp (*C. portucalensis*), Cf (*C. farmeri*), Pm (*P. mirabilis*), Mm (*M. morganii*).
