# Supplementary material for: Genomic analysis of antimicrobial resistance and virulence among gram-negative bloodstream isolates from Lebanon
Source: Microbiol Spectr. 2026 Jun 17;14(7):e00503-26. doi: 10.1128/spectrum.00503-26 (PMC13340248; doi:10.1128/spectrum.00503-26)
Supplement: Fig. S2 — In silico detection of antibiotic resistance genes in E. coli (Ec1-Ec3, Ec5, Ec7-Ec12, Ec14-Ec18), K. pneumoniae (Kp1-Kp5), Citrobacter spp. (Cp, Cf), and P. mirabilis (Pm). Resistance gene classes are labeled as follows: A, aminoglycosides; An, antiseptics; C, chloramphenicol; F, fosfomycin; M, macrolides; Q, quinolones; R, trimethoprim; S, sulfonamides; T, tetracyclines. [file spectrum.00503-26-s0002.pdf]

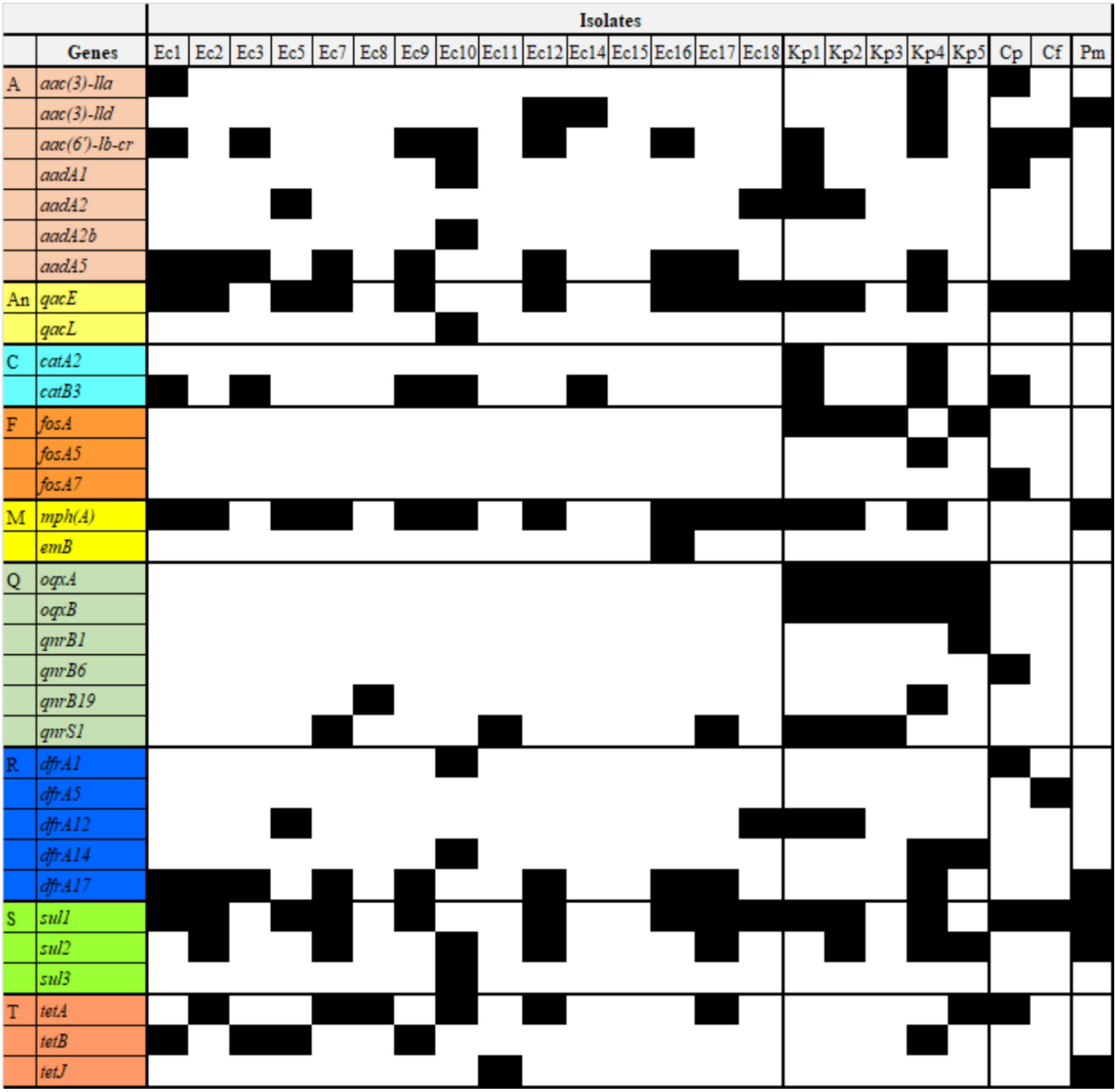
Isolates
Genes
Ec1
Ec2
Ec3
Ec5
Ec7
Ec8
Ec9
Ec10
Ec11
Ec12
Ec14
Ec15
Ec16
Ec17
Ec18
Kp1
Kp2
Kp3
Kp4
Kp5
Cp
Cf
Pm
A
aac(3)-IIa
aac(3)-IId
aac(6')-Ib-cr
aadA1
aadA2
aadA2b
aadA5
An
qacE
qacL
C
catA2
catB3
F
fosA
fosA5
fosA7
M
mph(A)
emB
Q
oqxA
oqxB
qnrB1
qnrB6
qnrB19
qnrS1
R
dfrA1
dfrA5
dfrA12
dfrA14
dfrA17
S
sul1
sul2
sul3
T
tetA
tetB
tetJ

Fig. S2 *In silico* detection of antibiotic resistance genes in *E. coli* (Ec1–Ec3, Ec5, Ec7–Ec12, Ec14–Ec18), *K. pneumoniae* (Kp1–Kp5), *Citrobacter* spp. (Cp, Cf), *and P. mirabilis* (Pm). Resistance gene classes are labeled as follows: A, aminoglycosides; An, antiseptics; C, chloramphenicol; F, fosfomycin; M, macrolides; Q, quinolones; R, trimethoprim; S, sulfonamides; T, tetracyclines.
