# Supplementary material for: Genomic analysis of antimicrobial resistance and virulence among gram-negative bloodstream isolates from Lebanon
Source: Microbiol Spectr. 2026 Jun 17;14(7):e00503-26. doi: 10.1128/spectrum.00503-26 (PMC13340248; doi:10.1128/spectrum.00503-26)
Supplement: Fig. S3 — Virulence determinants detected in E. coli, K. pneumoniae, C. portucalensis, C. farmeri, P. mirabilis, and M. morganii isolates. (Unk: unknown) [file spectrum.00503-26-s0003.pdf]

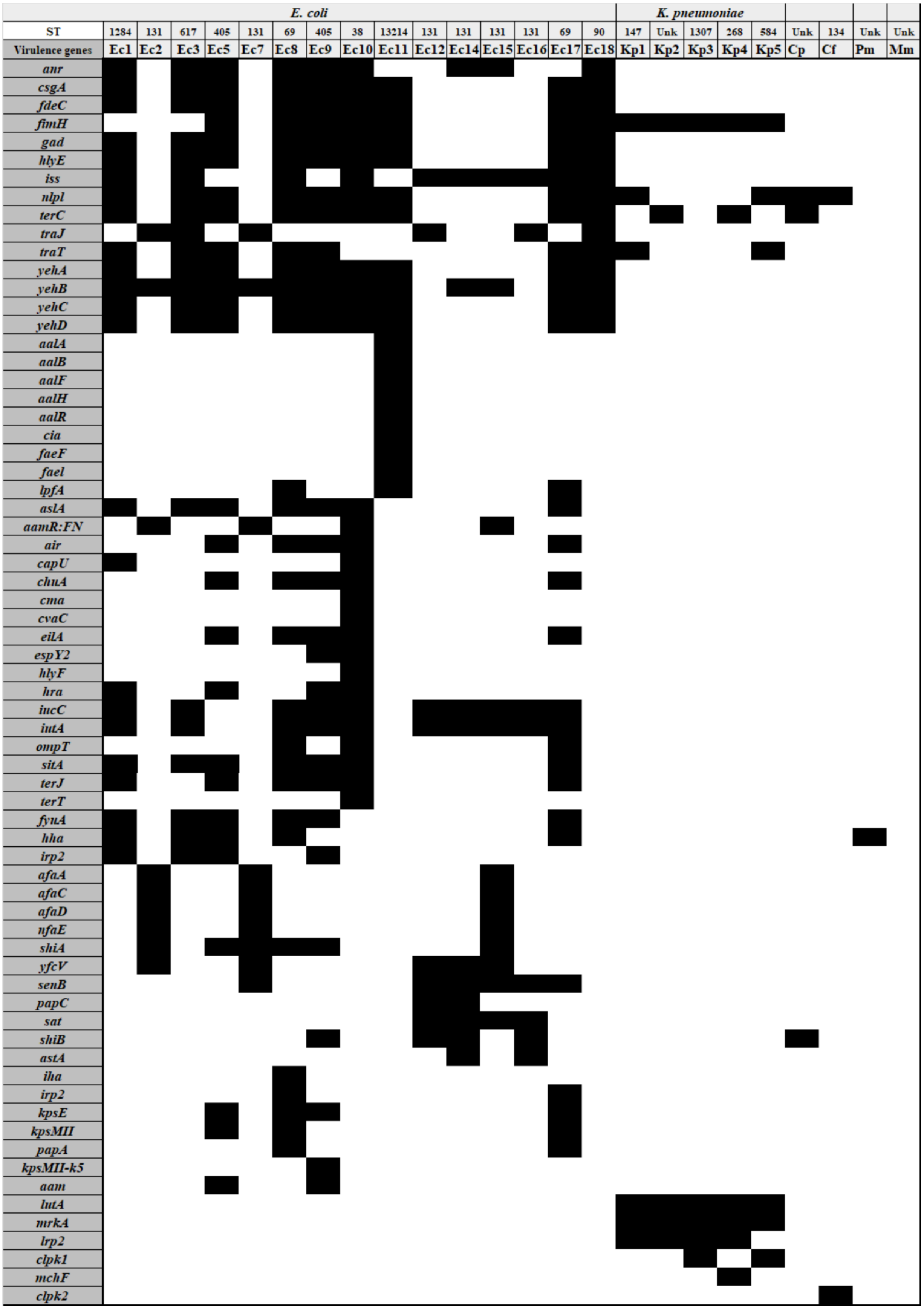
E. coli
K. pneumoniae
ST
1284
131
617
405
131
69
405
38
13214
131
131
131
131
69
90
147
Unk
1307
268
584
Unk
134
Unk
Unk
Virulence genes
Ec1
Ec2
Ec3
Ec5
Ec7
Ec8
Ec9
Ec10
Ec11
Ec12
Ec14
Ec15
Ec16
Ec17
Ec18
Kp1
Kp2
Kp3
Kp4
Kp5
Cp
Cf
Pm
Mm
anr
csgA
fdeC
fimH
gad
hlyE
iss
nlpI
terC
traJ
traT
yehA
yehB
yehC
yehD
aalA
aalB
aalF
aalH
aalR
cia
faeF
faeI
lpfA
aslA
aamR:FN
air
capU
chuA
cma
cvaC
eilA
espY2
hlyF
hra
iucC
iutA
ompT
sitA
terJ
terT
fyuA
hha
irp2
afaA
afaC
afaD
nfaE
shiA
yfcV
senB
papC
sat
shiB
astA
iha
irp2
kpsE
kpsMII
papA
kpsMII-k5
aam
lutA
mrkA
lrp2
clpk1
mchF
clpk2

Fig. S3 Virulence determinants detected in *E. coli*, *K. pneumoniae*, *C. portucalensis*, *C. farmer*i, *P. mirabilis*, and *M. morganii* isolates. (Unk: unknown)
