# Supplementary material for: Genomic analysis of antimicrobial resistance and virulence among gram-negative bloodstream isolates from Lebanon
Source: Microbiol Spectr. 2026 Jun 17;14(7):e00503-26. doi: 10.1128/spectrum.00503-26 (PMC13340248; doi:10.1128/spectrum.00503-26)
Supplement: Table S1 — NCBI accession numbers corresponding to the whole-genome sequences of each isolate. [file spectrum.00503-26-s0004.pdf]

Table S1

NCBI accession numbers corresponding to the whole-genome sequences of each isolate.

Isolates	Kmer-Finder	Accession Number	NCBI Name
Ec1	*Escherichia coli*	JAVVME000000000	CB-2023-1
Ec2	*Escherichia coli*	JAVVMF000000000	CB-2023-2
Ec3	*Escherichia coli*	JAVVMD000000000	CB-2023-3
Ec5	*Escherichia coli*	JAVVMH000000000	CB-2023-5
Ec7	*Escherichia coli*	JAVVMJ000000000	CB-2023-7
Ec8	*Escherichia coli*	JAVVMB000000000	CB-2023-8
Ec9	*Escherichia coli*	JAVVMK000000000	CB-2023-9
Ec10	*Escherichia coli*	JAVVML000000000	CB-2023-10
Ec11	*Escherichia coli*	JAVVMC000000000	CB-2023-11
Ec12	*Escherichia coli*	JAVVMM000000000	CB-2023-12
Ec14	*Escherichia coli*	JAVVMN000000000	CB-2023-14
Ec15	*Escherichia coli*	JAVVMO000000000	CB-2023-15
Ec16	*Escherichia coli*	JAVVMP000000000	CB-2023-16
Ec17	*Escherichia coli*	JAVVMQ000000000	CB-2023-22
Ec18	*Escherichia coli*	JAVVMR000000000	CB-2023-23
Kp1	*Klebsiella pneumoniae*	JAVVMU000000000	CB-2023-17
Kp2	*Klebsiella pneumoniae*	JAVVMV000000000	CB-2023-18
Kp3	*Klebsiella pneumoniae*	JAVVMW000000000	CB-2023-19
Kp4	*Klebsiella pneumoniae*	JAVVMX000000000	CB-2023-20
Kp5	*Klebsiella pneumoniae*	JAMVMY000000000	CB-2023-21
Cp	*Citrobacter portucalensis*	JAVVMG000000000	CB-2023-4
Cf	*Citrobacter farmeri*	JAVVMI000000000	CB-2023-6
Mm	*Morganella morganii*	JAVVMT000000000	CB-2023-13
Pm	*Proteus mirabilis*	JAVVMS000000000	CB-2023-24
