# Supplementary material for: Genomic analysis of antimicrobial resistance and virulence among gram-negative bloodstream isolates from Lebanon
Source: Microbiol Spectr. 2026 Jun 17;14(7):e00503-26. doi: 10.1128/spectrum.00503-26 (PMC13340248; doi:10.1128/spectrum.00503-26)
Supplement: Table S2 — Genome size (bp), number of contigs ({greater than or equal to}1000 bp), read count, N50 (bp), and GC content (%) are shown for each of the 24 BSI isolates. [file spectrum.00503-26-s0005.pdf]

Table 2. Sequencing and assembly metrics for all isolates.

Genome size (bp), number of contigs (≥1000 bp), read count, N50 (bp), and GC content (%) are shown for each of the 24 BSI isolates.

Isolate	Genome Size (bp)	# of Contigs (≥1000bp)	Read Count	N50 (bp)	GC %
Ec1	4919119	82	792741	126444	50.82
Ec2	5102405	44	785420	341707	50.75
Ec3	5029023	67	631700	211058	50.69
Ec5	5292089	64	342413	135936	50.56
Ec7	5209374	52	928140	378845	50.57
Ec8	5367980	56	1049585	207028	50.64
Ec9	5198938	57	678263	240814	50.7
Ec10	5260115	53	752746	302596	50.54
Ec11	4877888	39	528091	222638	50.75
Ec12	5207701	40	488142	419896	50.74
Ec14	5376794	71	409900	232445	50.69
Ec15	5307766	63	413135	211794	50.64
Ec16	5220602	66	1178279	229990	50.72
Ec17	612787	36	514303	320036	50.72
Ec18	759132	36	719960	350993	50.74
Kp1	1013986	52	1551122	504758	56.93
Kp2	644325	44	621950	268793	57.11
Kp3	1361717	53	1232765	697290	57.02
Kp4	504972	79	463650	247913	56.76
Kp5	1298228	29	468545	460098	57.35
Cp	5381208	33	725862	589250	51.72
Cf	5742512	70	910882	224125	53.08
Mm	3855952	12	452226	2417483	51.07
Pm	729501	30	373926	257850	38.88
